# Preferences for Follow-Up Procedures among Patients Lost to Follow-Up after Smoking Cessation Intervention among Therapists—An Interview Study

**DOI:** 10.3390/ijerph21060726

**Published:** 2024-06-03

**Authors:** Sanne Wärjerstam, Camilla Dew-Hattens, Mette Rasmussen, Berit Lilienthal Heitmann, Rie Raffing, Hanne Tønnesen

**Affiliations:** 1 WHO Collaborating Centre, Clinical Health Promotion Centre, Research Unit for Dietary Studies, The Parker Institute, Bispebjerg & Frederiksberg Hospital, University of Copenhagen, DK-2400 Copenhagen, Denmark; 2 National Institute of Public Health, University of Southern Denmark, DK-1455 Copenhagen, Denmark; 3 Section for General Practice, Department of Public Health, University of Copenhagen, DK-1172 Copenhagen, Denmark

**Keywords:** smoking cessation intervention, follow-up rate, loss to follow-up, patient preferences, therapist preferences

## Abstract

Achieving high follow-up rates after smoking cessation interventions (SCIs) is a general challenge. The aim of this study was to identify preferences among patients and therapists for improving follow-up rates and to assess smoking status at 6 months among patients lost to follow-up. From the Danish STOPbase for Tobacco and Nicotine, which collects data on SCI across health care, 20 representative patients lost to follow-up by routine procedures were identified together with 11 therapists. All participated in individual semi-structured phone interviews, which for patients also included 6-month smoking status. Deductive and inductive analyses were performed. Four themes emerged from the analyses with several subthemes, all regarding contacts. Both patients and therapists preferred to intensify the follow-up process by boosting it with additional attempts and using voice messages, e-mail and/or SMS, calling at specified times of the day and avoiding calls from unknown numbers. In addition, some patients mentioned that they were busy or were not carrying their mobile devices at the time of a call as a barrier. Some therapists mentioned that barriers could include an expectation of relapse, but also a poor mental state, the time of day and patient fear of public systems. Among the patients originally lost to follow-up, 35% (95% CI 16%–59%) experienced continuous smoking cessation for 6 months, and the overall national rate was 22% (21.6–23.3%). In conclusion, both patients and therapists preferred intensified follow-up. The 6-month smoking status for patients lost to follow-up seemed to be similar to that of the routinely followed-up patients. These findings will be examined experimentally in a larger study.

## 1. Introduction

Determining the long-term efficacy of smoking cessation interventions (SCIs) is a global challenge. Ideally, the standard for follow-up rates should be approximately 80% [1]. However, response rates are often lower, even in the most recent SCI studies [2,3,4,5,6,7]. Almost two decades ago, an important step was taken to introduce six international criteria, mainly based on the intention-to-treat approach, to secure standardized measurement and reporting of outcomes in randomized SCI trials [8]. The criteria could also be useful for other study designs, like cohorts. To avoid overestimating an SCI effect, smoking status among patients lost to follow-up should be recorded as smokers unless they died or emigrated. However, this criterion might underestimate the successful quitting rate, especially in studies with a low follow-up rate.

It was not possible to identify previous interview studies on the follow-up preferences of staff and patients after smoking cessation among those lost to follow-up. In 2009, a systematic review evaluated the effect of different strategies to improve the response to postal and electronic questionnaires based on 481 and 32 studies, respectively [9]. Only two of the studies concerned SCI follow-up [10,11]. Unfortunately, the review did not include follow-up by interviews, face-to-face, online or by telephone call. 

Like in the United Kingdom [12], Denmark has a widespread coverage of STOP Units in primary health care. Since 2001, they have reported to the STOPbase for Tobacco and Nicotine for national and local monitoring of the effect to ensure high-quality SCI. Today, more than 200,000, or about 95%, of smokers and users of other tobacco and nicotine products have given informed consent to the registration and follow-up. The manual-based follow-up procedure includes a structured telephone interview with at least three telephone reminders [12]. Despite the fact that more than 90% are attempted, only about 60% complete the six-month follow-up [13]. There is a large variability in follow-up rates across the country, from 25% to almost 100%. Thus, it seems doable to improve the follow-up rate. 

Therefore, the primary aim of this study was to identify preferences, facilitators and barriers to improving the follow-up rate after intensive SCI among patients lost to follow-up and therapists. A secondary aim was to report the successful quitting rate among those patients.

## 2. Materials and Methods

### 2.1. Research Team

The research team consisted of six people, two of whom were interviewers. SW, a registered nurse, PhD student and experienced therapist for almost 40 years, interviewed the therapists. CDH, the project collaborator with a Master of Science, interviewed the patients. The interviewed therapists and patients received the information via invitation mail. The other four individuals were scientific experts: RR, an anthropologist with expertise in qualitative methods; BLH, a professor with expertise in observation and intervention research; MR, experienced in tobacco epidemiology with a Master of Science and PhD; and HT, a professor, chief physician, and Doctor of Medical Science.

### 2.2. Study Design

**Figure 1:**
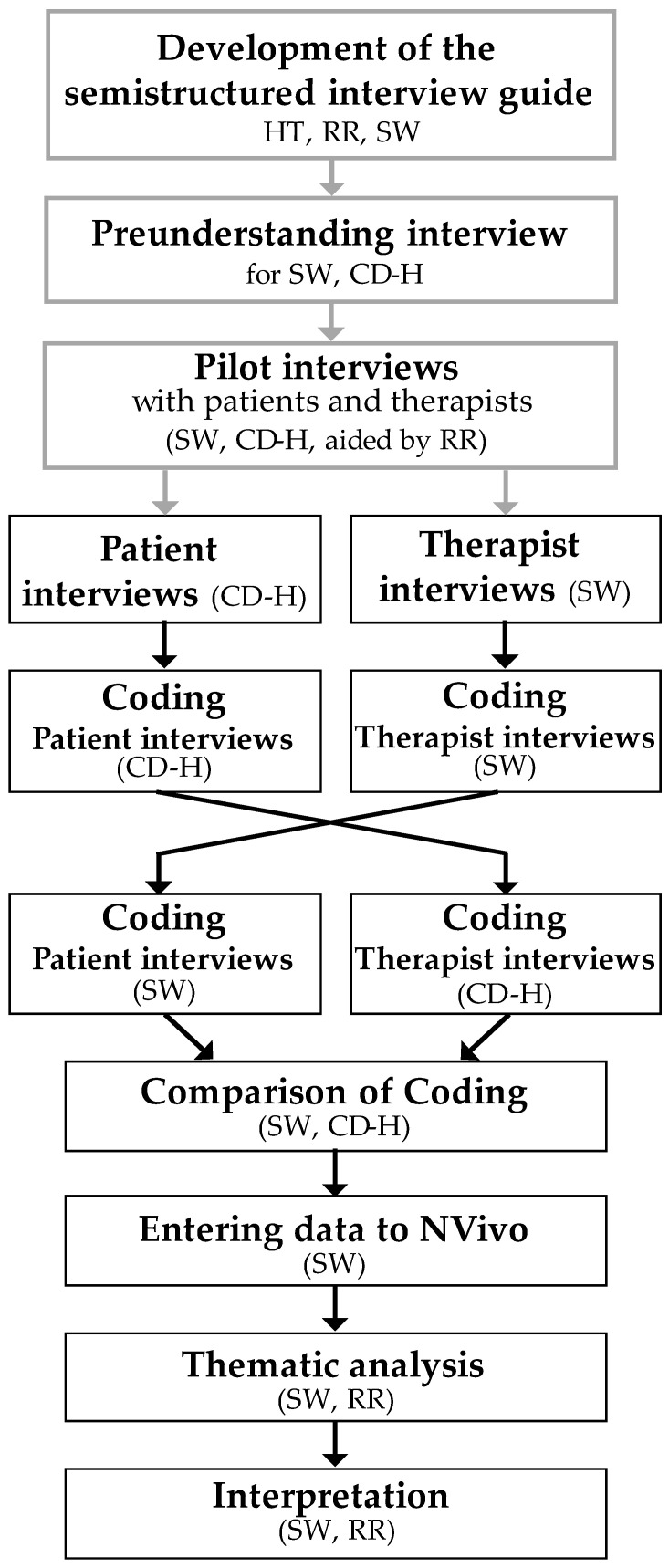
Interview process, coding, analysis and interpretation.

The phenomenological method was determined to be the best method to answer the research question because it offers opportunities to understand human experiences without interpreting the respondents’ answers and thus is effective for exploring the preferences of patients and therapists [13] (Figure 1). The first step in using the phenomenological method is, to the best of one’s abilities, to identify one’s own preunderstanding. To achieve this goal, a member of the research team (RR) performed a recorded preunderstanding interview with each of the interviewers (SW and CDH) to uncover preunderstandings prior to data collection. Two preunderstandings were identified, namely, the continued smoking of those who could not be reached at the routine follow-up and the preferred time of day for follow-up.

### 2.3. Recruitment, Participants and Consent

The sampling was purposive and aimed to ensure geographical representativeness for both patients and therapists from STOP Units delivering SCI services across the country. To properly investigate the goal of improving patient follow-up rates presented a methodological paradox: how do you contact patients who are characterized as lost to follow-up?

The group of patients lost to follow-up at the 6-month follow-up was characterized by providing informed consent for registration and follow-up when they entered the SCI and by not having responded to at least four telephone calls on different days, including at least one call in the evening hours taking place from the fifth to the seventh month after the planned stop date (see Appendix C).

On average, approximately eight to fifteen participants are needed in qualitative interviews to achieve saturation, and twelve participants have been suggested to reach major as well as minor themes [14]. Due to the foreseen difficulty of contacting the patient group, a buffer was included.

Patient information about the interviews on preferences for improvement of the follow-up rate and an individual follow-up for smoking status was sent to 48 patients lost to 6-month follow-up via a protected e-mail (the national electronic box system) together with contact information for the STOPbase. Subsequently, patients received a telephone call and/or text messages. The first 20 responding patients volunteered to participate and received an appointment for a telephone interview with the CDH. The patients provided their original consent for a 6-month follow-up for smoking status and further consented to participate in the interview.

The patients were representative of geography, sex and age for patients in the STOPbase. They had participated in the smoking cessation program in 2023, and the interviews took place in December 2023. The patient data were collected four months after the loss of the routine 6-month follow-up, i.e., at the end of the seventh month following the planned day of quitting.

A priori, all the therapists affiliated with the STOPbase had received information about the project on their theme day and provided informed consent prior to the interview. Therapists who generally had a high (at least 80%) follow-up rate and therapists who had a low (55% or below) follow-up rate were included; thus, three of the six therapists with a high follow-up rate and eight of the twenty-four therapists with a low follow-up rate were interviewed by SW, for a total of eleven therapists.

### 2.4. Setting

The data were collected by phone with the interviewer in the office of the research institution or working from home. The interviewee could be reached anywhere where they decided to pick up their phone, and it is unknown if they were alone or together with other persons during the interview.

### 2.5. Data Collection

An interview guide was designed with semi-structured questions for the patient and therapist interviews. After the presentation, an introduction and opening questions, the patients and staff were asked about their wishes for contact for follow-up and options for improving the follow-up procedure, possible barriers and whether they knew why the routine contact for follow-up was not successful. The interviews ended with a summary and request for additional comments (see Appendix A and Appendix B). Data on smoking status were also collected from the patients according to the guidelines of the STOPbase. A pilot interview with the patient and therapist was conducted by telephone. During these pilot interviews performed by SW and CHD, a third person from the research team (RR) participated to ensure the quality of the interviews, confirming that the interview guide was followed and that a correct understanding of the answers was provided. Since these interviews did not give rise to changes in the interview guides, they were included in the main study. During the interviews, detailed field notes were written by hand, and the answers were repeated for the respondents at the end of the interviews to ensure that the answers were understood correctly. Each interview took approximately 15 to 30 min to complete. Each patient and therapist were interviewed once. At the end of the interviews, the patients were asked about their smoking status six months after the program was completed according to the manual and follow-up [2].

### 2.6. Analysis

All interview field notes, both from patients and from therapists, were coded separately by the two interviewers (SW and CDH), and coding differences were discussed to achieve a consensus. Subsequently, the data were entered into N’Vivo^®^ and recoded (SW) and analyzed for themes (SW and RR). The data from the patients were collected both deductively and inductively. In this way, both predefined themes of relevance to the research question and themes directly from the data were identified.

A coding tree was not developed due to the nature of the data collection with field notes for the responses, and for the same reasons, quotations were not presented. Participants were not asked to provide feedback on the findings. Major and minor themes were identified. During the interviews, substantial notes were obtained, but as the interviews were not recorded, representative quotes could not be presented in the results.

### 2.7. Ethics

The STOPbase has been approved by the Danish Data Protection Agency (P-2021-900) and further considered by the Scientific Ethical Committee of the Capital Region (685 27), which did not have further comments. All patients provided informed consent for the registration and collection of follow-up data via the STOPbase when undergoing SCI. In addition, specific consent was obtained for the interview (see Section 2.3 above). After all information was received, the therapists verbally provided permission to participate in the study.

## 3. Results

This study led to the identification of four themes: facilitators for successful follow-up, preferred methods of contact, preferred methods of follow-up, preferred contact time and barriers to successful 6-month follow-up. These were common for patients and therapists. Please see the detailed description below.

Both groups preferred to intensify the follow-up process by boosting the follow-up procedure, adding voice messages, mail and/or SMS; calling at specified times of the day; and avoiding calls from an unknown number. In addition, some patients mentioned being busy or not carrying their mobile devices at the time of the call as barriers. The therapists mentioned increasing the number of follow-up attempts and/or beginning follow-up earlier as facilitators, while their expected barriers included primarily patients’ smoking relapse but also a poor mental state of the patients at the time of call, time of day and the possibility of fear of public systems among patients.

### 3.1. Patients

The characteristics of the 20 interviewed patients at six months of follow-up after SCI are shown in Table 1.

Overall, answers and reflections were reported in groups of persons with similar responses as follows: “few”, *n* = 3–4; some, *n* = 5–10; “most” *n* = 11–20 (See Table 2 below).

Concerning facilitators for successful follow-up, the subthemes were mixed. Some patients mentioned being followed up by mail and by SMS, and others reported that they did not know. A few preferred being prepared for contact (in addition to providing informed consent).

Additionally, regarding the subthemes of preferred choice of contact and time, some patients preferred telephone, email, and SMS contact. In addition, some patients preferred specific time periods, such as the afternoon, dinnertime, one hour after work, late afternoon and afternoon/evening, while a few had no preferences. A few specified preferred times, such as 2, 3 and 6 pm, while others simply preferred sometime during the day.

The agreement was greater concerning returning telephone calls from therapists in response to a voice message. Most patients mentioned that they would return a call, while a few were uncertain or indicated that they would not return a call. However, most patients would not return a call from an unknown number.

Greater agreement was obtained concerning barriers. Most patients reported that receiving a call from an unknown number was a barrier, and some mentioned that being busy, having a bad conscience and not carrying a mobile phone hindered their ability to answer the phone. Some other patients did not know why the attempt to contact them failed. A few patients mentioned barriers to responding, such as low energy, not wanting to answer the phone in public places, working issues and the therapist not being available when they attempted to return the call.

### 3.2. Therapists

The characteristics of the 11 interviewed therapists involved in the six months of follow-up of patients after SCI (Table 2).

**Table 2:** Characteristics of the 11 therapists interviewed.

Number of Participants	11
Gender, women	10
Education	
Longer (long or medium)	11
Regions	
North Jutland	2
Central Denmark	2
Southern Denmark	1
Zealand	0
Capital Region of Denmark	6

The analyses revealed that most of the patients and therapists agreed on four common themes: facilitators for successful follow-up, preferred method of contact, preferred contact time and barriers to successful 6-month follow-up. The related responses and reflections from the 11 therapists were grouped as follows based on the number of therapists who reported similar answers: “few”, *n* = 3; “some”, *n* = 4–5; “most” *n* = 6 or more (Table 3).

The subthemes were also mixed among the therapists. Concerning facilitators for successful follow-up, some therapists mentioned that patients should be well informed about the contact (in addition to providing informed consent). Most therapists mentioned that the time of day when calling for follow-up was important. However, they did not agree on the specific preferred times and suggested nonspecific times such as daytime, during working hours, as late as possible, early, late, not during evenings and at the same time as the intervention course. Most patients also suggested a specific time after 4 p.m., e.g., 4:30–5 p.m., 5 p.m., 5–7 p.m. and 7–7:30 p.m.

Furthermore, most of the therapists reported that they would like to follow up with the patients more frequently than required based on the guidelines of the STOPbase, which require follow-ups at the end of the intervention and after 6 months, ranging from 5–7 months. They mentioned that adding a follow-up at 1–3 months and/or approximately 4 months might improve the 6-month follow-up response.

Concerning the number of calls made before giving up on the follow-up, some therapists mentioned three to four calls or four calls during working hours and after 4 pm, both of which are very close to the guidelines of the STOPbase. To improve successful follow-ups, most therapists mentioned the utility of telephone calls and an SMS if the patient did not answer; some others would leave a voice message. Some therapists noted that the patients themselves returned phone calls, though the frequency of this ranged from 5–10% (most) to 50–100% (a few). A few therapists indicated that their patients did not return phone calls.

Concerning barriers, most therapists mentioned barriers such as patients relapsing, lacking surplus energy and receiving calls from an unknown number. Some mentioned that patients may have been in a poor mental state, had mental illness or a bad conscience due to relapse, were afraid of public systems or felt they were being called at an inconvenient time of day. A few therapists stated that patients may have had a new telephone number.

**Table 3:** Similarities and differences in themes and subthemes across patients and therapists. For subthemes among the 20 patients, answers were classified based on the number of patients with similar responses, as follows: few, 3–4; some, 5–10; most, 11–20. For the 11 therapists, the results were classified based on the number of therapists with similar responses as follows: few, 3; some, 4–5; most, 6–11. Overall, values denoted as ‘–’ indicated less than few.

	Patients	Therapists
	*n* = 20	*n* = 11
Identified facilitators for successful follow-up		
Sending email	Some	-
Sending SMS	Some	-
Improved preparation of patients regarding contact	Few	Some
Following up earlier than 6 months	-	Most
Did not identify facilitators	Some	-
Preferred method of contact		
Telephone	Some	Most
SMS	Some	Most
Mail	Some	-
Telephone message	-	Some
Preferred calling time		
A specific time (such as 2, 3 or 6 pm)	Few	Most
A specific period (after 4 pm)	-	Most
Specific periods (such as afternoon, dinnertime, evening)	Some	-
During daytime	Few	-
No preferred time	Few	-
Identified barriers for successful 6-month follow-up		
Therapists using an unknown phone number	Most	Most
Patients having a bad conscience	Some	Some
Patients lacking surplus energy	Few	Most
Patients being busy	Some	-
Patients not carrying a mobile phone	Some	-
Patients not answering their phone in public places	Few	-
Patients having a work issue	Few	-
Therapists not available	Few	-
Patients having relapsed	-	Most
Patient in a poor mental state or suffering from mental illness	-	Some
Inconvenient time of day	-	Some
Patient fear of public systems	-	Some
Patient changing telephone number	-	Few
Did not identify barriers	Some	-

The preferences of the subthemes showed broader variation, especially for barriers (Table 3). Overall, both groups stated preferences regarding what and what not to do in practice, mainly pointing towards intensification of the follow-up procedures with a greater number of contact efforts and specific methods to achieve a higher follow-up rate.

Finally, regarding smoking status 6 months after SCI, 7/20, corresponding to 35% [95% CI: 16–59%], reported 6 months of continuous smoking cessation, while the figures in the national STOPbase were 2186/9756, corresponding to 22% [22–23%] (Table 4). Despite the overlap of the two confidence intervals indicating similar effects, the sample size of the group lost to follow-up was too small to draw any conclusions.

## 4. Discussion

Four themes emerged in this interview study to identify preferences among patients and therapists to improve follow-up rates after SCI. The four themes concerned facilitators, time of contact, methods of contact and barriers for both groups, all of which had several subthemes and were important for practice. Both groups preferred to intensify the follow-up process by strengthening the follow-up procedure through the addition of voice messages, e-mail and/or SMS, extending calls at specified times of day and avoiding calls from unknown numbers. In addition, some patients mentioned being busy or not carrying their mobile devices at the time of calls as barriers. The therapists mentioned a need to increase the number of follow-up attempts and/or beginning the follow-up earlier, while they also highlighted barriers such as patient relapse, a poor mental state of the patient at the time of the call, calls made at inconvenient times of the day and patient fear of public systems.

Several studies have evaluated different strategies to increase the follow-up of different screenings and interventions, especially for postal and electronic questionnaires, as previously reviewed by Edwards and coworkers [9]. Follow-up for smoking status and other health behaviors was evaluated in a randomized trial comparing mailed questionnaires and telephone interviews, which showed higher response rates for telephone interviews [15]. In contrast, the results of telephone interviews from another study of 272 of 500 respiratory patients lost to follow-up showed that smoking was especially common, but respiratory symptoms were similar to those in the routine follow-up group [16]. In this present study, the group of patients who were originally lost to follow-up explained that the main reasons for not completing the follow-up were that they were busy or had forgotten, while only a very small proportion did not want to participate. These results are consistent with the findings of our study on patient and staff preferences.

To date, it has not been possible to identify other studies evaluating preferences among patients and staff to improve the follow-up rate after SCI. The overall agreement in this study between the two groups regarding intensified follow-up is important for practice. Despite the disagreement among the two groups about subthemes, e.g., preferences of specific times of day for follow-up calls, the results of the interviews created a basis to optimize the follow-up procedure, for instance, by introducing a new possibility to align with patients on individual dates and methods for follow-up that fit both the patients’ and their therapists’ needs. Likewise, both groups mentioned avoiding calls from an unknown telephone number, which is an action item that can immediately be implemented. In practice, these adjustments may slightly increase the work for therapists, and the effects of intensified follow-up solutions need to be evaluated in a real-life setting.

The results showed that approximately one-third of the interviewed patients had successfully quit smoking 6 months after the SCI, which was surprising, as the expectation from the preunderstanding interviews was that relapse was a major reason for loss to follow-up. It seems counterintuitive that those lost to follow-up had a high continuous quit rate, as for decades, missing follow-up data have been considered to hinge on unchanged smoking status, as described in the Russel criteria [8]. However, the main reason for loss to follow-up in this study was that the patients did not answer the phone calls. In the interviews, the patients expressed a willingness to participate in the routine follow-up. Nevertheless, for several reasons, this was not possible (Table 3), identified barriers), and during the interviews, patients responded to the usual data collection used in the STOPbase. This group may therefore have responded differently from other groups who refused to answer mailed forms or to online data collection [9]. In addition, this group was too small to draw conclusions, but we may hypothesize that similar results could be obtained when investigating this in a future larger study.

### 4.1. Strengths, Bias, and Limitations

A strength of this study is that it included both patients as end users and their therapists. Additionally, the research group succeeded in contacting a patient group that was lost to follow-up and hence had not been previously successfully contacted. However, telephone interviews may involve a certain degree of bias, as the interviewer cannot observe positive or negative body language reactions. Conversely, smoking status was self-reported, and some of the patients may have misreported their status. A strength of this current study, however, is that self-reported data were collected during an interview instead of from patients filling out a questionnaire in isolation [15]. It would have been preferable to use biomarkers for validation. Equipment could even have been sent home for collection of saliva or urine, or the sampling could have been conducted at the STOP Unit, which would, however, have required an extra physical visit. Other possibilities could include providing patients with equipment for online monitoring via CO measurements or oximetry. These validations increase costs, and the reliability of self-validations is sparsely reported as follow-up after SCI.

The adherence to the recommendations regarding pre-interview conduct can be considered a strength, as one preunderstanding was identified [17]. Methodologically, independent dual coding was a strength. The therapists’ answers were coded inductively, while the patient data were coded using inductive and deductive methods due to the nature of the responses. However, by collecting notes and keywords in handwritten form, the interviewer may omit certain words and nuances.

Coding in the same way is preferable, but using both inductive and deductive approaches may ensure that predefined themes and themes from the data answer the research questions. A coding tree was not presented because the answers in the patient group were often binary yes/no and otherwise short answers. These present findings may have been biased by the fact that the interviewers were bilingual (Danish–Swedish and Danish–English), which could have affected the language in the notes differently. However, the interviews of both groups were coded independently and separately, and the coding was compared to assess agreement. The agreement with the material content was high.

### 4.2. Perspectives

From a patient perspective, the current results are important; in particular, to base the reporting of the effect on a higher follow-up rate in the future and a reduction in inconvenience through the use of a known telephone number of the therapist and better preparation and alignment about the specific time and day of telephone calls during the 6-month follow-up.

From a clinical perspective, the STOPbase has already implemented an online possibility for local agreement on the follow-up day and time. Furthermore, therapists may have to change the assumption that patients are continuous smokers if they do not respond to follow-up, and it would also be beneficial for patients to avoid this negative expectation.

At the societal level, it is important to obtain high follow-up rates of SCI and thereby more reliable information on successful quitting, as improved quit rates are more cost-effective than lower quit rates, with all else being equal. In particular, civil servants, policy planners, public health researchers and politicians need this information for the relevant estimations used for deciding free access to programs for quitting, smoking bans and other health-promoting initiatives.

Further research should be conducted to evaluate the effect of the preferred intensified follow-up process in a larger study and incorporate more patient perspectives. It could also include an evaluation of the intensive follow-up after AI programs.

For practical reasons, it is recommended that patients agree on the day and time of follow-up and are informed about the telephone number used for calling. Both items are to be included in the updated guidelines for follow-up in the STOPbase, and the impact of follow-up should be evaluated afterwards.

## 5. Conclusions

Patients and therapists agreed on four common themes and identified several subthemes focused on improving the follow-up process and creating the basis for new guidelines. These results must be tested in a future study. Additionally, the expectation that almost all patients who are lost to follow-up have relapsed may not be valid.

## Figures and Tables

**Table 1:** Characteristics of the 20 patients included in the interviews and the participants in the National STOPbase in 2023, presented as numbers (%) or medians (ranges).

	Study Participants	The STOPbase
Number of patients	20	9756
Gender, women, *n* (% of total)	11 (55%)	(52%)
Age, years, median [range]		
Women	57 [36–75]	55 [12–88]
Men	56 [17–71]	55 [12–96]
Education, *n* (% of total)		
Long and medium education	5 (25%)	(45%)
Short education	15 (75%)	(49%)
Missing data	0	(6%)
Occupation, *n* (% of total)		
In business	(55%)	(38%)
Not in business	(40%)	(59%)
Missing data	(5%)	(2%)
Region, *n* (% of total)		
North Jutland	5 (25%)	1038 (11%)
Central Denmark	4 (20%)	2744 (28%)
Southern Denmark	3 (15%)	2708 (28%)
Zealand	3 (15%)	1062 (11%)
Capital Region of Denmark	5 (25%)	2204 (23%)

**Table 4:** Successful quitting at the 6-month follow-up of the 20 patients who were originally lost to follow-up and were included in the study and all 9756 persons from the national STOPbase in 2022 with follow-up in 2023 [15]. The 95% confidence intervals for proportions were estimated using the Wilson score method.

	Study	95% CI	STOPbase	95% CI
Number of participants	20		9756	
Intervention completed (≥75% meeting adherence)	11 (55%)	32–76%	6109 (63%)	61.6–63.6%
Successful 6-month follow-up (STOPbase guidelines)	0 (0%)		5752 (59%)	58.0–59.9%
Successful 6-month follow-up (this interview study)	20 (100%)		-	
6-month continuous smoking cessation	7 (35%)	16–59%	2186 (22%)	21.6–23.3%

## Data Availability

The data from the STOPbase are available upon reasonable request and with great consideration and respect for the privacy and anonymity of the participants.

## Appendix A. Interview Guide for Patients

“Hello, my name is Camilla, and I am calling in connection with the smoking cessation course you have completed in/at XXX from the to the d. X.

We have sent you a message that we are contacting you because we want to be better at following up on these courses. It will take approximately 10 min, and I would like to inform you that your participation is voluntary, so you can stop the interview at any time without any consequences to you. Your name will be anonymized in the notes from the interview and in the analysis, and it will not be possible to recognize your statements in the material we develop based on the interview. Is that okay with you?”

Preliminary questions:

How have you been doing since you took the course?

Has your advisor has tried to contact you about following up on the course?

Preferences:

If you could choose freely, how would you prefer to be contacted by counsellors who follow up on your smoking cessation?

What time of day do you prefer to be contacted?

How do you feel about calling back on…

- A voice message.
- A missed call from a number you do not know.

Options:

Where do you think there are opportunities to connect with more participants when we want to follow up after a smoking cessation course?

Causes:

What barriers do you think could prevent us from succeeding with follow-ups?

Xxx (the unit) has stated that they have attempted to follow up on your course on xx, xx, xx and xx. Do you know why they did not get in touch with you?

“To summarize what you have said and to make sure that I have understood it correctly, I will briefly repeat some of your answers”.

Transition:

“I would like to hear slightly more about the progress toward your goal after the smoking cessation course”.

Questionnaire: (Continuously successful quitting and smoke-free for the past 14 days)

Stopped: “… In addition, how amazing it is that you have reached your goal”.

Not stopped but stepped down: “It is great that you have been able to step down, and I will keep my fingers crossed that you achieve what you want”.

Not stopped: “I’m keeping my fingers crossed that you achieve what you want.

## Appendix B. Interview Guide: Therapists

“Hello, my name is Sanne, and I am calling from the STOPbase. You can hear that I am not Danish, but I hope you understand me; otherwise, you are very welcome to ask for clarification.

We have sent you an email saying that we would contact you regarding the follow-up of patients who have participated in a smoking cessation course. With respect to STOPbase, we want to understand more about how we can achieve better results after 6 months of follow-up. This process will only take 10–15 min, and I would like to inform you that your participation is voluntary; you cannot recognize your answers in the results, and you can stop the conversation at any time without any consequences. Is it okay to move on?”

Preliminary questions:

I am curious to hear how you follow-up today after 6 months on your device?

Preferences:

If you could choose freely, how do you think it is best to contact participants during follow-up?

When do you think it is best to contact them?

Options:

What do you think could help you get in touch with even more people through follow-up?

Causes:

What do you think is preventing you from connecting with your participants?

Is there anything else that affects contact with participants?

Do these reasons match your own experience of why it can be difficult to connect with participants at follow-up?

What do you think could help you get in touch with even more people through follow-up?

To summarize what you have said and that I have understood correctly, I will briefly repeat your answers.

Before we finish, I have one more specific question:

Preferences for Closed Questions:

Do you find that participants call back when you have attempted to follow-up but have not been in contact?

How often?

Thank you so much for your time; it was a great help.

## Appendix C. The STOPbase Manual for 6-Month Follow-Up Interviews

One systematic follow-up must be conducted for each patient. This follow-up must be carried out 6 months after the planned stop date, within a time interval of ±1 month.

By systematic follow-up, we mean the following:

- Contact must be made by telephone.
- All patients who provided written consent and agreed to be contacted (Q11 on the basic form) were contacted.
- Each patient was contacted at least four times.

Follow-up must be performed by calling:

To ensure that the results registered in the STOPbase are comparable, all follow-ups must be performed by telephone interviews. It is not permitted to send out the forms to the patients and ask them to return the answered questionnaire.

Each patient must be contacted at least four times on different days, including at least one call in the evening hours (after 5 p.m.). If the patient cannot be reached after four calls, the follow-up can be considered lost. In that case, the follow-up must be registered on the form “Reason for lack of follow-up”, under the item “The patient does not answer despite at least 4 phone calls”. In the protected self-registration system for the STOP Units, a function is built that helps to keep track of when a patient has been called and has not answered the phone.

As a starting point, follow-up must be performed for all patients:

Follow-up must be performed for all patients who have given their written consent (click on the basic form) and who agree to the question “May the therapist or an external consultant contact you later to determine how things are going?” (Q11 on the basic form). Patients who have not completed the intervention or have started smoking again must also be contacted.

The participants who have answered no to be contacted in connection with a follow-up will automatically be sorted from the follow-up list found in the self-testing environment. All participants who appear on the follow-up list must therefore be called 6 months after their cessation.

Recommendations:

The STOPbase recommends that follow-up be carried out by the national Quitline (Stoplinien) or a similar professionally competent organization/person who can advise patients. If Stoplinien makes the call, they have the option of registering the patient for a new intervention in case of relapse. By using the Stoplinien, you also ensure that the follow-up is as uniform as possible, and thus that the data quality is as high as possible.

Based on the questions on the follow-up form, the STOPbase also recommends that it is not the specific adviser with whom the patient has attended the intervention who carries out the follow-up because it may be more difficult to respond if the therapists themselves conduct the follow-up interviews.
